# Diagnosing ALS: the Gold Coast criteria and the role of EMG

**DOI:** 10.1136/practneurol-2021-003256

**Published:** 2022-01-06

**Authors:** Martin R Turner, UK MND Clinical Studies Group, Ammar Al-Chalabi

**Affiliations:** Nuffield Department of Clinical Neurosciences, University of Oxford Medical Sciences Division, Oxford OX3 9DU, UK

**Keywords:** ALS, EMG, motor neuron disease

In September 2019, a group of international neurologists gathered in Gold Coast, Australia, to deconstruct the diagnostic process for amyotrophic lateral sclerosis (ALS) and to try to simplify it. A proposal for revised diagnostic criteria emerged^1^ (box 1) and the initial experience of their application has been positive.^2–5^ The diagnosis remains fundamentally clinical, and it is timely to reflect on the adjunctive role of electromyography (EMG).

Box 1 The Gold Coast criteria for the diagnosis of amyotrophic lateral sclerosis

1. *
**Progressive*** motor impairment documented by history or repeated clinical assessment, preceded by normal motor function,

**AND**

1. The presence of **upper** * and **lower** † motor neurone dysfunction in at least **ONE body region** ‡, with:
  - upper and lower motor neurone dysfunction noted in the same body region if only one region is involved,
  - or lower motor neurone dysfunction in at least TWO body regions,

**AND**

1. Investigations§ excluding other disease processes.

*Upper motor neurone dysfunction implies **at least one** of the following:

- Increased deep tendon reflexes, including the presence of a reflex in a clinically weak and wasted muscle, or spread to adjacent muscles.
- Presence of pathological reflexes, including Hoffman sign, Babinski sign, crossed adductor reflex, or snout reflex.
- Increase in velocity-dependent tone (spasticity).
- Slowed, poorly coordinated voluntary movement, not attributable to weakness of lower motor neurone origin or Parkinsonian features.

†Lower motor neurone dysfunction in a given muscle requires **either** :

- Clinical examination evidence of muscle weakness and muscle wasting, or
- EMG abnormalities that must include both:
  - Evidence of chronic neurogenic change, defined by large motor unit potentials of increased duration and/or increased amplitude (with polyphasia), and motor unit instability regarded as supportive but not obligatory evidence, and
  - Evidence of ongoing denervation, including fibrillation potentials or positive sharp waves, or fasciculation potentials.

‡Body regions are defined as bulbar, cervical, thoracic and lumbosacral. To be classified as an involved region with respect to lower motor neurone involvement, there must be abnormalities in TWO limb muscles innervated by different roots and nerves, or ONE bulbar muscle, or ONE thoracic muscle, either by clinical examination or by electromyography (EMG).

§The appropriate investigations depend on the clinical presentation, and may include nerve conduction studies and needle EMG, MR or other imaging, biofluid studies, or other modalities as clinically indicated.

Although motor neurone disease (MND) is undeniably clinically heterogeneous, there is a unifying molecular pathology in 97% of cases, namely cytoplasmic phosphorylated aggregates of the 43 kDa transactive response DNA-binding protein, TDP-43. Since Charcot’s pivotal clinical observations of the simultaneous occurrence of upper motor neurone (UMN) and lower motor neurone (LMN) involvement defining the the most common phenotype of ALS, a broader spectrum of involvement is now recognised clinically and pathologically.^6^ At both LMN and UMN clinical extremes, the rate of disease progression is notably slower, but there is no simplistic relationship between the site of onset, the combination of UMN versus LMN signs or rate of disease progression. The historical term progressive muscular atrophy was used to describe the extreme of clinical LMN involvement, but histopathological^7^ and neuroimaging^8^ studies have shown that such cases have subclinical corticospinal tract and wider brain involvement, and the modern use of the term ‘LMN-predominant ALS’ reflects this. The other extreme is also a spectrum of ‘UMN-predominant ALS’ in which LMN signs are not clinically obvious until a few years after symptom onset. A minority of these cases, and less than 3% of all MND, can be confidently demarcated as primary lateral sclerosis by virtue of a dramatically slower rate of progression associated with marked spasticity as well as lack of LMN involvement.^9^ The clinicopathological overlap of ALS with frontotemporal dementia has further extended Charcot’s definition, so that ALS may now be considered a *system* degeneration variably penetrating the motor cortex and its spinal and cerebral connectome.

The original diagnostic criteria defined at El Escorial, Spain, in 1990 and their subsequent 1998 revision at Airlie House, USA, and in 2006 at Awaji-shima, Japan, employed categories but were created very much with therapeutic trials in mind. Categories were based on the number of body regions (bulbar, cervical, thoracic and lumbosacral) with simultaneous UMN as well as LMN signs. The terms used in the various iterations, included some or all of: ‘suspected’, ‘possible’, ‘probable’, ‘probable laboratory-supported’ and ‘definite’ ALS. These categories have been shown to have little independent prognostic value^10^ and patients with MND deemed to have ‘insufficient’ UMN signs clinically were denied entry to trials, despite similar rates of disability progression as ‘probable’ or ‘definite’ cases. Individuals frequently die from MND never having evolved from their ‘suspected’, ‘possible’ or ‘probable’ categories. It is biologically interesting how little clinical evolution there is between these categories, despite a wide range of disability progression rates common to all of them. Speculatively, the proportions of UMN versus LMN involvement might reflect an individual’s premorbid nervous system architecture to some extent. Most problematic of all, however, was the often psychologically distressing consequences for patients in being told they had anything other than ‘definite’ ALS, implying persistent diagnostic doubt where there was usually none.

EMG has been unrivalled to date in providing evidence of subclinical LMN involvement, although with persistent debate as to what changes are pathognomic for neurodegenerative denervation. Importantly however, EMG is far from 100% sensitive and highly operator dependent. The sensitivity shortfall is most often apparent in cases of UMN-predominant ALS, particularly those with bulbar onset. Historically (but no longer usefully) termed progressive bulbar palsy, such cases are commonly found in women aged over 70 years. Symptoms may remain relatively confined to the bulbar territory for more than a year before wider generalisation. There is often a lack of tongue as well as limb EMG findings. In such cases, over-reliance on EMG as a diagnostic test for MND leads to avoidable diagnostic delay. This may impact timely care planning, for example, gastrostomy, access to licensed disease-modifying drugs like riluzole and trials of new candidate drugs.

The Gold Coast group recognised rapid developments in new potential biomarkers of subclinical UMN degeneration, for example, blood neurofilament concentrations, diffusion tensor imaging, transcranial magnetic stimulation; and LMN degeneration, for example, muscle ultrasound detection of subclinical fasciculation. Such biomarkers might lead to further refinement of these criteria. In the longer-term, a TDP-43 assay might even form the basis for wider population screening. For now, the immediate hope is that the Gold Coast criteria will remove the distress and confusion caused by the historical diagnostic category terms. The new criteria still have therapeutic trials very much at the forefront of thinking. Replacing site of symptom onset, only one factor influencing overall prognosis, with a multiaxial classification based on rate of progression and the pattern of progression of disability might ultimately provide a more meaningful basis for stratification.

For the individual with progressive weakness, the diagnosis is ‘ALS’, or ‘not ALS’. EMG remains valuable in providing evidence of clinically occult LMN involvement but is an adjunct to a history and examination-led diagnostic process. Finally, a cornerstone of good medical practice is that any diagnosis, but especially a life-shortening one, should be followed by an outline of proposed care. We suggest that the diagnosis of ALS should be conveyed by a clinician with knowledge of that plan.

**Further reading**

- Vucic S, Ferguson TA, Cummings C, Hotchkin MT, Genge A, Glanzman R, *et al*. Gold Coast diagnostic criteria: implications for ALS diagnosis and clinical trial enrollment. *Muscle & Nerve*. 2021.
